# Structural Insights into Reelin Function: Present and Future

**DOI:** 10.3389/fncel.2016.00137

**Published:** 2016-05-27

**Authors:** Fanomezana M. Ranaivoson, Sventja von Daake, Davide Comoletti

**Affiliations:** ^1^Child Health Institute of New Jersey, Robert Wood Johnson Medical School, Rutgers UniversityNew Brunswick, NJ, USA; ^2^Department of Neuroscience and Cell Biology, Robert Wood Johnson Medical School, Rutgers UniversityNew Brunswick, NJ, USA; ^3^Department of Pediatrics, Robert Wood Johnson Medical School, Rutgers UniversityNew Brunswick, NJ, USA

**Keywords:** Reelin, ApoER2, VLDLR, brain development, cortical layers, structure-function

## Abstract

Reelin is a neuronal glycoprotein secreted by the Cajal-Retzius cells in marginal regions of the cerebral cortex and the hippocampus where it plays important roles in the control of neuronal migration and the formation of cellular layers during brain development. This 3461 residue-long protein is composed of a signal peptide, an F-spondin-like domain, eight Reelin repeats (RR1–8), and a positively charged sequence at the C-terminus. Biochemical data indicate that the central region of Reelin binds to the low-density lipoprotein receptors apolipoprotein E receptor 2 (ApoER2) and the very-low-density lipoprotein receptor (VLDLR), leading to the phosphorylation of the intracellular adaptor protein Dab1. After secretion, Reelin is rapidly degraded in three major fragments, but the functional significance of this degradation is poorly understood. Probably due to its large mass and the complexity of its architecture, the high-resolution, three-dimensional structure of Reelin has never been determined. However, the crystal structures of some of the RRs have been solved, providing important insights into their fold and the interaction with the ApoER2 receptor. This review discusses the current findings on the structure of Reelin and its binding to the ApoER2 and VLDLR receptors, and we discuss some areas where proteomics and structural biology can help understanding Reelin function in brain development and human health.

## Introduction

Disruptions of the autosomal recessive *Reelin* gene were identified two decades ago to be responsible for the *reeler* phenotype in mice strains originated from Edinburgh and Orleans (D’Arcangelo et al., 1995; Hirotsune et al., 1995). The Edinburgh homozygous mutant *reeler* mouse displays a complete loss of transcription of the gene (D’Arcangelo et al., 1995) whereas the Orleans strain expresses a Reelin protein that lacks a C-terminal portion (Hirotsune et al., 1995; D’Arcangelo et al., 1997; de Bergeyck et al., 1997). Despite the different genomic abnormalities, both strains are characterized by specific neurological phenotypes including tremors, ataxia, cerebellar hypoplasia and malformation of cellular layers throughout the brain (Falconer, 1951; Angevine and Sidman, 1961; Caviness and Rakic, 1978; Pinto-Lord et al., 1982; Rakic and Caviness, 1995; Lambert de Rouvroit and Goffinet, 1998, and others). The involvement of Reelin in layer formation in brain cortical structures was extensively investigated (for an excellent review, see D’Arcangelo, 2014). It is now well established that during embryonic brain development, Reelin has a crucial role in controlling the radial migration of neurons, allowing them to reach their appropriate positions in laminated structures such as the cerebral cortex, the hippocampus or the cerebellum (Lambert de Rouvroit and Goffinet, 1998). Control of neuronal migration and layer formation is achieved by expression and secretion of Reelin by specific sub-types of cells, namely by Cajal-Retzius cells in marginal regions of the cerebral cortex and the hippocampus (D’Arcangelo et al., 1995; Ogawa et al., 1995; Del Río et al., 1997; Nakajima et al., 1997; Schiffmann et al., 1997; Alcantara et al., 1998), or by granule cell precursors localized in the external granule layer of the embryonic cerebellum (D’Arcangelo et al., 1995; Miyata et al., 1996; Alcantara et al., 1998). Additionally, small level of *Reelin* expression have been detected in deeper layers of the cerebral cortex (Yoshida et al., 2006; Uchida et al., 2009; Hirota et al., 2015). It is thought that secreted full length Reelin directs the migration of neurons in contact with these regions, whereas proteolytic fragments (see below), which diffuse towards deeper cortical layers, may target local neurons and initiate their polarization and their radial migration (Utsunomiya-Tate et al., 2000; Kubo et al., 2002; Jossin et al., 2007). Reelin was also shown to influence neurite formation in early postnatal brain (Del Río et al., 1997; Olson et al., 2006; Matsuki et al., 2010; Nichols and Olson, 2010) and to impact synapse formation and function in late postnatal and adult brain (Borrell et al., 1999; Liu et al., 2001; Rice et al., 2001; Qiu et al., 2006; Iafrati et al., 2014). At least some of these effects come into play through the association of Reelin with two well-known receptors of the low density lipoprotein receptor (LDLR) superfamily: the apolipoprotein E receptor 2 (ApoER2) and the very-low-density lipoprotein receptor (VLDLR) (D’Arcangelo et al., 1999; Hiesberger et al., 1999; Trommsdorff et al., 1999; Benhayon et al., 2003).

Following the initial observation that *REELIN* mRNA levels are reduced in patients with schizophrenia (Impagnatiello et al., 1998), several investigators reported a deficiency in Reelin expression in different groups of psychiatric subjects, including those with bipolar disorder (Knuesel, 2010). The reduction in Reelin expression occurs most likely through epigenetic mechanisms that affect promoter methylation (Veldic et al., 2004; Grayson et al., 2006) although evidence for genetic association between schizophrenia and *REELIN* polymorphisms also exists in patient subpopulations (Goldberger et al., 2005; Wedenoja et al., 2008). Furthermore, Reelin has been reported to suppress schizophreniform symptoms in mice (Ishii et al., 2015). In addition to schizophrenia, reduced expression and *REELIN* polymorphisms have been reported in some groups of autistic patients (De Rubeis et al., 2014).

This review discusses the architecture and three-dimensional structure of Reelin, highlighting the elements that are involved in the interaction with ApoER2 and VLDLR. We will discuss the proteolysis of Reelin and we will suggest areas of exploration that, in our opinion, will provide valuable information of the structural and functional mechanisms underlying the biological function of Reelin.

## Structure of Reelin

**Figure 1:**
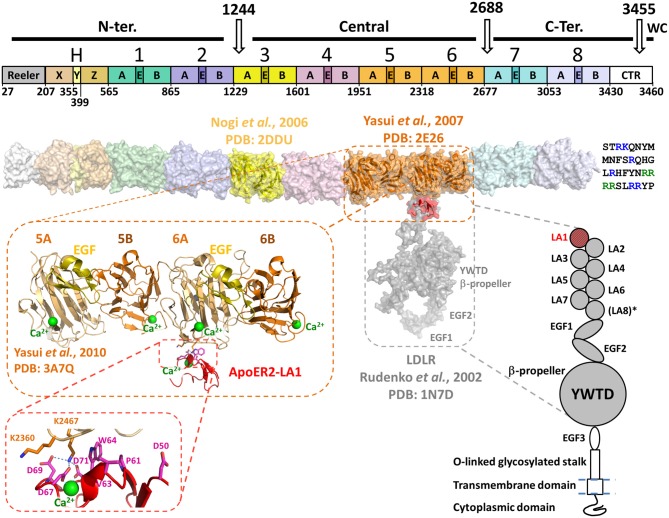
**Domain organization and structural model of Reelin in association with apolipoprotein E receptor 2 (ApoER2) or very-low-density lipoprotein receptor (VLDLR).** Top diagram shows the domain organization of Reelin. Three *in vivo* proteolysis sites (white arrows) are represented on the top of the diagram, highlighting the N-terminal (N-ter.), Central, C-terminal (C-ter.) and the WC cleavage products. The Reelin repeats are numbered (1–8) and their sub-domain composition (subrepeats A and B separated by an epidermal growth factor (EGF)-like (E) domain) is marked. On the left side of RR1 the Reeler domain and the domains X, Y and Z (fragment “H”) are marked. The white box on the right side (C-terminally RR8) represents the conserved fragment of the CTR. The boundaries of each domain and RR are indicated on the bottom of the diagram and are identified by numbers corresponding to the mouse Reelin sequence (Uniprot : Q60841) and are adapted from Ichihara et al. (2001). Below the bar diagram, a three-dimensional model of Reelin (surface representation) was assembled using the established crystal structures of RR3 (yellow, PDB: 2DDU) and RR5–6 (orange, PDB: 2E26) and homology-models of each other RR and the X, Y and Z domains. The template used for the Reeler domain model was the N-terminal domain of F-spondin (PDB: 3COO). The homology models were built with the Swiss-model server^1^ and the RR models were positioned relatively to each other to best reproduce the inter-repeat interface observed in the crystal structure of RR5–6. The CTR is represented at the C-terminus of the Reelin model as a single-letter amino acid sequence. The basic residues are highlighted in blue, and the WC cleavage recognition sequence (RRRR) is green. Schematic representation of the LDLR-like receptor is shown on the lower right side of the figure. The domain organization of ApoER2 and VLDLR is similar to the architecture of LDLR, with the exception of an extra LDLR class A (LA) module not found in the latter (LA8*). Both ApoER2 and VLDLR receptors contain eight LA modules, three EGF-like domains, a unique β-propeller formed by YWTD (or LDLR class B) repeats separating EGF2 and EGF3. They also contain an O-linked glycosylation portion (stalk) upstream a single-pass transmembrane domain and a C-terminal cytoplasmic domain. No structure of large fragments of ApoER2 or VLDLR are currently available, thus the structure of the equivalent fragment of LDLR (PDB: 1N7D) was used to illustrate the binding with Reelin on the three-dimensional model (Rudenko et al., 2002). LDLR LA1 was overlaid with ApoER2 LA1 (red) in complex with RR5–6 (PDB: 3A7Q). The overall three-dimensional model uses the same color-code as the domain organization diagrams, for both partners. The crystal structure of RR5–6 in complex with ApoER2 LA1 is shown in more detail in the zoomed-in inset to highlight the folding of this Reelin fragment, and the location of the interfacing residue with ApoER2. The Reelin sub-repeats and EGF-like domain are distinguished by their color: A in light orange, B in dark orange and EGF in yellow. The Ca^2+^ ions characteristic to Reelin sub-repeats and the receptor LA module are represented as green spheres. A detailed view of the major interfacing residues is illustrated in the inset, showing the critical interaction pattern established by Reelin Lys2467 with ApoER2 Asp69, Asp71 and Asp67.

Reelin is a ~440 kDa secreted glycoprotein (~386 kDa with ~18 putative N-linked glycosylation sites) expressed from a large genomic section (~450 kb spanned by 65 exons) located on chromosome 5 in mouse and 7 in humans. Mouse and human Reelin have ~94.2% and ~87.2% sequence identity at the amino acid and DNA levels, respectively (DeSilva et al., 1997). From the N- to the C-termini, Reelin is composed of a Reeler domain similar to the N-terminal domain of F-spondin, followed by a unique region named “H” (~300 amino acids), subdivided in three subdomains termed X, Y and Z (Ichihara et al., 2001) and eight Reelin-specific repeats followed by a highly basic region. Each Reelin repeat (RR) is ~350–390 amino-acid residues long, divided into two homologous sub-repeats (A and B) separated by an epidermal growth factor (EGF) domain of about 35 amino-acid residues (Figure 1). The eight RRs are structurally similar, with an average sequence identity in the range of 83.5–87.9% when comparing the same RR of Reelin homologs from 167 species. Interestingly, the amino acid identity of the eight RR in the same molecule is in the range of 36.4–39.7% (Manoharan et al., 2015), suggesting that albeit the identity is sufficient to maintain similar tertiary structure, these domains are likely to be functionally different (e.g., interact with different receptors).

Although the overall structure of Reelin has never been reported, individual structures of selected RRs were solved by macromolecular crystallography. The crystal structure of RR3 (Nogi et al., 2006, PDB ID: 2DDU) revealed that each sub-repeat of Reelin is composed by 11 β-strands organized in two antiparallel β-sheets that are arranged in parallel, forming a jelly-roll folding similar to the carbohydrate-binding module (CBM) of other unrelated proteins, and to the “galactose-binding domain-like” superfamily. One of the features of a RR is a compact “horseshoe-like” conformation, where sub-repeats A and B make extensive interfacial contacts, burying ~1600 Å^2^ of solvent-accessible area, including a loop protruding from the concave side of sub-repeat A (Nogi et al., 2006). This loop bulges from the middle of the fifth strand (strand “H”) of the β-sheet at the concave side and is found in all aligned Reelin sub-repeats A. The EGF-like domain that separates the two sub-repeats in the primary structure lies on the lateral side of the tertiary arrangement where it interacts with both sub-repeats A and B. A stretch of ~14 amino-acids termed “bacterial neuraminidase repeat” (BNR) or “Asp-box” is found in all Reelin sub-repeats in close proximity to the EGF-like domain. This motif (X-X-S/T-X-D/N-X-G-X-X-W, where X can be any amino acid) has been found in a few other mammalian proteins such as Sialidase1–3 proteins (Gaskell et al., 1995) and forms a β-hairpin that seems to have a structural rather than a functional purpose (Copley et al., 2001).

The β-sheets of the sub-repeats are curved, creating a concave and a convex side of the jelly-roll shape where a Ca^2+^ ion is bound at the convex side of RR3B, but also in all four sub-repeats in the RR5–6 Reelin fragment (Yasui et al., 2007, PDB ID: 2E26). The importance of this bound Ca^2+^ is further emphasized by the finding of a similar calcium-binding site in other structurally related proteins, like in the CBM domains (Nogi et al., 2006) or the Cleaved Adhesin domain family (Ganuelas et al., 2013).^1^

Together with the crystal structure of RR3, single particle electron tomography was used to image the four-repeats-containing Reelin fragment RR3–6 (Nogi et al., 2006). In addition to the compactness of each individual RR, repeats RR3 through RR6 appeared to be in close contact with each other, leading to a roughly straight particle. This particle was solved as a flattened rod-shaped three-dimensional map, with a maximum dimension of approximately four times the longest dimension of a single repeat. The structural continuity between RRs was analyzed in further detail with the crystal structure of RR5–6 (Yasui et al., 2007). The two repeats were related to each other by an almost perfect translational symmetry, and the inter-repeat interface buried solvent-accessible area of ~1500 Å^2^, mainly composed by hydrophobic contacts, suggesting a stable intramolecular conformation of the two repeats.

## *In Vivo* Proteolysis of Reelin

*In vivo*, proteolysis of the mature protein occurs, over time, at two positions, within RR3 but close to the interface with RR2, and between RR6 and RR7. By antibody mapping, the fragmentation pattern of Reelin predicts the generation of three major proteolytic fragments (the so-called N-terminal, central and C-terminal fragments). The N-terminal cleavage site has been determined to occur between Pro-1244 and Ala-1245 within RR3 (Koie et al., 2014). Consistently, Reelin-P1244D mutant became protease resistant and had a longer biological effect compared to wild type (WT) Reelin (Koie et al., 2014). The C-terminal cleavage site of WT Reelin, between RR6 and RR7 was also recently identified between Ala2688 and Asp2689 (^2685^RSPA/DAG^2691^) (Sato et al., 2016). A Reelin mutant, in which Asp2689 is replaced by Lys appears to be resistant to C-terminal cleavage when incubated with culture supernatant of cerebellar granular neurons. Finally, bioinformatics analysis of the C-terminal Reelin sequence indicates the presence of a Furin recognition proteolysis site [R-X-(R/K)-R] between residues 3452 and 3455 (^3452^RRRR^3455^/SLRRYP^3461^). The presence of this cleavage site and the release of the last six amino acids were confirmed experimentally and named WC (Within the C-Terminal Region [CTR]) site (Kohno et al., 2015). Although the exact function of this cleavage is currently unknown, sequence conservation among mammals suggests a critical function in the mammalian brain. Taken together, these data indicate that cleavage of Reelin may play a critical role in regulating the duration and range of Reelin signaling in the developing brain.

## The Reelin Receptors

Reelin binds with similar affinity to cell surface receptors VLDLR and ApoER2, two members of the LDLR family. These receptors are differentially expressed in different organs, tissues, and cell types (Trommsdorff et al., 1999). Reelin binding to ApoER2/VLDLR receptors activates intracellular Src family kinases (SFKs), which in turn phosphorylate the adaptor protein Disabled-1 (Dab1) at specific tyrosine residues (Arnaud et al., 2003; Suetsugu et al., 2004). Although binding to Reelin triggers common signal transduction mechanisms, they mediate diverse biological functions (D’Arcangelo et al., 1999; Hiesberger et al., 1999; Benhayon et al., 2003).

Structurally, both receptors are composed of an extracellular domain of ~800 amino acids, followed by a single transmembrane and an intracellular domain (Figure 1). The architecture of both receptors is very close to the prototypical LDLR (Rudenko et al., 2002), with seven or eight “LDLR class A” (LA) domains followed by three EGF modules and a unique YWDT β-propeller domain inserted between EGF2 and EGF3 (Figure 1).

A variety of protein-protein interaction assays such as protein pull-down, surface plasmon resonance and isothermal titration calorimetry assays were used to evaluate and quantify the interaction of Reelin to either receptor (Andersen et al., 2003; Yasui et al., 2007, 2010). These experiments determined that RR3–6 contained the necessary binding elements to associate with ApoER2/VLDLR receptors (Jossin et al., 2004) and that the minimal binding cassette seems to be the RR5–6 fragment. On the receptor side, it was shown that the LA-containing regions of ApoER2 and VLDLR were necessary to bind RR5–6 (D’Arcangelo et al., 1997; Koch et al., 2002; Andersen et al., 2003). In particular, the first LA module (LA1) was necessary for the Reelin interaction providing a dissociation constant (K_D_) in the nanomolar range. Interestingly, according to the reported K_D_ values, supplemental LA domains appeared to modulate the affinity towards RR5–6. Between the two receptors, ApoER2-LA1 appeared to have higher affinity to RR5–6 than VLDLR-LA1 (Andersen et al., 2003; Yasui et al., 2010).

The crystal structure of the [RR5–6:ApoER2-LA1] complex revealed that the LA1 module interacts essentially with RR6A, close to the RR5–6 inter-Repeat interface (Yasui et al., 2010). Therefore, the observed essential role of RR5 in solution for the receptor association is likely to be indirect. Additionally, the high affinity interaction contrasts with the small interface area (696 Å^2^) observed in the crystal structure, suggesting that other portions of Reelin or the receptor may contribute to the interaction. Indeed, a multivalent binding mode is usually observed for the known ligand interactions involving LA modules in the LDLR family (Blacklow, 2007). For example the Receptor-Associated Protein (RAP), an endoplasmic reticulum escort protein for LDLR-like proteins, is capable of binding LA pairs rather than single LA modules (Andersen et al., 2000, 2001; Fisher et al., 2006).

A Ca^2+^ ion, characteristically bound to LA modules, is found in the ApoER2-LA1 structure, providing important structural basis for the observed Ca^2+^-dependency of the Reelin-receptor association (D’Arcangelo et al., 1999; Andersen et al., 2003). However, this ion-dependency may also result from the Reelin-bound Ca^2+^ described above (Nogi et al., 2006). Overall, the interaction between RR5–6 and the ApoER-LA1 is in accord with the canonical mode of interaction between LA modules and their protein partners as described by Blacklow (2007). This interaction involves a lysine (equivalent to RR5–6 Lys2467) or an arginine in interaction with three LA aspartate residues that are involved in the Ca^2+^-coordination, e.g., Asp67, Asp69 and Asp71 in ApoER2-LA1. Additionally, an aromatic side chain (equivalent to LA1 Trp64) stacks the aliphatic groups of the lysine side chain for a proper positioning for the interaction with the aspartates. Interestingly, the two structures of RR5–6 alone (Yasui et al., 2007) or in complex with the LA1 module of ApoER2 (Yasui et al., 2010) were almost perfectly superimposable, indicating that the binding of the receptor did not induce major structural changes within the repeats and sub-repeats of the RR5–6 fragment (Figure 1).

The VLDLR and ApoER2 receptors appear to mediate at least some of the known functions of Reelin in the central nervous system; however, other receptors may be responsible for additional function in the brain or in peripheral organs. In fact, Reelin was also found to associate with EphB2 and EphB3 by co-immunoprecipitation of both endogenous proteins from brain lysates (Sentürk et al., 2011). Unlike lipoprotein receptors, EphB2 appears to bind the N-terminal region of Reelin. This region was also reported to bind other putative Reelin receptors such as the Cadherin-related neuronal receptor-1 or CNR1 and integrin α3β1 (Senzaki et al., 1999; Dulabon et al., 2000). However, the binding to these receptors have not been further confirmed and characterized.

## Future Perspectives

A great deal of information is currently available on the structure and function of Reelin in the developing brain. Nevertheless, many questions remain unanswered. For example, Reelin/VLDLR or ApoER2 interaction occurs in the central region of Reelin but the N-terminal portion of Reelin also influences ApoER2 and VLDLR function. This is indicated by the inhibition of Reelin activity by the CR-50 antibody, which binds to an epitope located between residues 251 and 407 of Reelin (Del Río et al., 1997; D’Arcangelo et al., 1997, 1999; Miyata et al., 1997), presumably through inhibition of Reelin multimerization (Utsunomiya-Tate et al., 2000; Kubo et al., 2002). Interestingly, residue Cys2101, located in the central fragment of Reelin was recently shown to be involved in Reelin dimerization (Yasui et al., 2011), indicating that the multimerization of Reelin is complex. Moreover, the Reelin poly-basic 32-residue long CTR (Figure 1) appears to be functionally significant for the ApoER2 or VLDLR signaling pathway (Nakano et al., 2007; Kohno et al., 2009) and for the structure and positioning of neurons in the developing and the postnatal cerebral cortex (Kohno et al., 2015). This fragment is extremely conserved in mammals, birds and reptiles (Nakano et al., 2007) and seems to be important for the efficient secretion of Reelin (D’Arcangelo et al., 1997; de Bergeyck et al., 1997) by maintaining the structural integrity of RR8 (Kohno et al., 2009). Together, these studies highlight the importance of other parts of Reelin that, whilst not directly involved in the receptor interaction, contribute to its function. At the molecular and structural levels, further analyses are clearly needed to fully understand the implication of these parts of Reelin, as well as of the other RRs that have not been characterized so far. Furthermore, emerging investigations indicate that alternative cell surface receptors, such as the EphB2 and EphB3, likely interact with other parts of Reelin. Kohno et al. (2015) observed that the fragment RR7–8 that does not bind to the LDLR-like receptors is able to bind to neuronal cell membrane as long as it includes an intact CTR.

### Final Considerations

It will be interesting to characterize the full-length Reelin structurally, alone, and bound to its canonical receptors. However, the large size, extensive N-linked glycosylation, multimerization, and potential intra-molecular flexibility, make this study difficult to complete using protein crystallography. A combination of lower resolution approaches, including small angle X-ray scattering (Rubio-Marrero et al., 2016) and cryo electron microscopy (EM; Zhou, 2008) are likely to be the methods of choice. Another fascinating element is the significance of the proteolysis of Reelin. The degradation pattern seems to be well established and it is currently thought to negatively modulate the function of Reelin. However, it is also possible that cleavage into various RR segments enable these smaller fragments to diffuse to more distant brain layers and to bind and activate currently unrecognized cell surface receptors. In either case, the dynamic regulation of these events is not well understood and it should be an area of intense research. A precise profiling of Reelin degradation over time will be necessary to understand Reelin function in health and disease. Outstanding work has been done to determine the N- and C-terminal cleavage sites, as well as the newly recognized WC site. However, it is likely that, *in vivo*, Reelin is further degraded into smaller fragments that can have currently unrecognized activities.

Finally, it was recently reported that full-length Reelin, but not its central fragment, is capable of activating Erk1/2 signaling, leading to increased p90RSK phosphorylation and the induction of immediate-early gene expression. Remarkably, because Erk1/2 activation is not mediated by the canonical signal transduction pathway, a non-canonical pathway that works during brain development must exist (Lee et al., 2014).

## Author Contributions

All authors have contributed to the writing of the manuscript. All authors listed, have made substantial, direct and intellectual contribution to the work, and approved it for publication.

## Conflict of Interest Statement

The authors declare that the research was conducted in the absence of any commercial or financial relationships that could be construed as a potential conflict of interest.

## Footnotes

^1^http://swissmodel.expasy.org
